# Genetic counseling during COVID‐19 pandemic: Tuscany experience

**DOI:** 10.1002/mgg3.1433

**Published:** 2020-08-03

**Authors:** Angelica Pagliazzi, Giorgia Mancano, Giulia Forzano, Fabiana di Giovanni, Giulia Gori, Giovanna Traficante, Achille Iolascon, Sabrina Giglio

**Affiliations:** ^1^ Medical Genetics Unit Department of Clinical and Experimental Biomedical Sciences "Mario Serio" University of Florence Florence Italy; ^2^ Medical Genetics Unit Meyer Children's University Hospital Florence Italy; ^3^ Department of Molecular Medicine and Medical Biotechnology University Federico II of Naples Naples Italy

**Keywords:** COVID‐19, genetic counseling, pandemic, telegenetics, telemedicine

## Abstract

**Background:**

COVID‐19 outbreak prompted health centres to reorganize their clinical and surgical activity. In this paper, we show how medical genetics department's activity, in our tertiary pediatric hospital, has changed due to pandemic.

**Methods:**

We stratified all our scheduled visits, from March 9th through April 30th, and assessed case‐by‐case which genetic consultations should be maintained as face‐to‐face visit, or postponed/switched to telemedicine.

**Results:**

Out of 288 scheduled appointments, 60 were prenatal consultations and 228 were postnatal visits. We performed most of prenatal consultations as face‐to‐face visits, as women would have been present in the hospital to perform other procedures in addition to our consult. As for postnatal care, we suspended all outpatient first visits and opted for telemedicine for selected follow‐up consultations: interestingly, 75% of our patients’ parents revealed that they would have cancelled the appointment themselves for the fear to contract an infection.

**Conclusions:**

Spread of COVID‐19 in Italy forced us to change our working habits. Given the necessity to optimize healthcare resources and minimize the risk of in‐hospital infections, we experienced the benefits of telegenetics. Current pandemic made us familiar with telemedicine, laying the foundations for its application to deal with the increasing number of requests in clinical genetics.

## Graphical abstract

In this paper, we show how medical genetics department's activity, in our tertiary pediatric hospital, has changed due to COVID‐19 pandemic. Given the necessity to optimize healthcare resources and minimize the risk of in‐hospital infections, we experienced the benefits of telegenetics. Current pandemic made us familiar with telemedicine, laying the foundations for its application to deal with the increasing number of requests in clinical genetics.

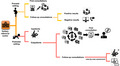

## 1 INTRODUCTION

In Italy, "genetic consultants," after graduating in medicine, acquire training and experience in genetic counseling through a specific residency program. Since virtually every aspect of the human figure and physiology is influenced by genetic information, it is not surprising that physicians who work in this field often must specialize further. Genetic subspecialties encompass almost all the different fields of the clinic, not only prenatal diagnosis, pediatric, and tumor genetics. The relationship between physician and patient has always been and remains a keystone for assistance and in the field of genetics it also takes on a greater purpose: the means by which data, diagnosis and treatment plans are collected, imply sharing for other members of the family. The relationship between patient and physician is, therefore, more demanding for both parties and must be based on mutual trust.

As of April 30th 2020, SARS‐CoV‐2 virus was accounted for 203591 infections and 27682 deaths in Italy (World Health Organization, https://www.who.int/emergencies/diseases/novel‐coronavirus‐2019/situation‐reports), making our country one of the worst affected. COVID‐19 outbreak has proven to be a rapidly evolving and complex situation: the fast spread of SARS‐CoV‐2 infection in Italy at the end of February led Italian government to set a nationwide lockdown starting on March 9th. Given the state of emergency, health centres were asked to reorganize clinical and surgical activities in order to reduce the risk of contamination within the hospital as well as to get prepared to admit the prospectively growing number of affected patients. In this unexpected scenario, national and regional authorities suggested to identify and perform only urgent and non‐deferrable procedures, suspending elective and postponable activity. In absence of standardized guidelines, each surgical (Cini et al., 2020) and clinical (Danese et al., 2020) department had to individually face their activities’ reorganization, in order to maintain as good as possible standard of care, to optimize healthcare resources and to reduce the risk of hospital‐acquired infection to lowest term.

Aim of this present paper is to show how, in a tertiary pediatric hospital, our medical genetics department's activity has changed due to COVID‐19 outbreak.

## 2 MATERIALS AND METHODS

We queried our prospectively maintained outpatient clinic database from March 9th through April 30th. We recorded patients’ personal data, type of consultation and clinical indication: in detail, we stratified our visits considering type of counseling (prenatal and postnatal), type of appointment (first or follow‐up visit), and, finally, type of setting (outpatient clinic or inpatient consultation). We, therefore, took note of how many and, more interesting, which type of consultations we decided to (1) maintain as face‐to‐face visit, (2) reschedule later in time or (3) switch to telemedicine consultation, trying to draw up as standardized as possible criteria. To test the appreciation of telemedicine, we sent an email survey to the families’ address, after the consultation.

During that period, every member of our hospital's medical staff was tested for SARS‐CoV‐2 virus, through both rapid serologic test and nasopharyngeal swab simultaneously, in order to reduce hospital‐acquired SARS‐CoV‐2 infections. Similarly, all patients, who had to be hospitalized for scheduled or emergency procedures, were tested just before admission, in order to exclude a SARS‐CoV‐2 contamination. Outpatients and their caregivers were not routinely tested for COVID‐19 but they underwent temperature screening with a thermal scanner at hospital entrance. For each child, only another person (parent/caregiver) was allowed to enter the hospital. In exceptional cases and after physician's specific request, both parents could be admitted to the hospital, for example, to sign informed consent. Lastly, all patients and all members of hospital staff were provided with surgical masks.

## 3 RESULTS

**Figure 1:**
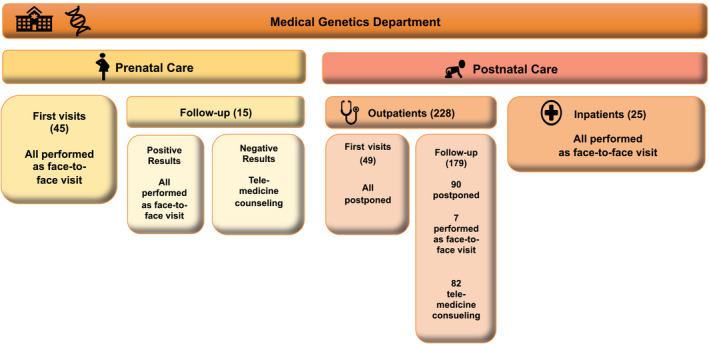
Schematic representation of genetic counseling activities during the COVID‐19 emergency

Out of 288 scheduled appointments, 60 were prenatal consultations and 228 were postnatal visits (Figure 1).

In our hospital, prenatal genetic consultation is generally programmed so to be performed along with other prenatal either invasive or non‐invasive procedures, providing women with a thorough and well‐scheduled prenatal diagnosis program. Given the non‐postponable nature of most of our prenatal consultations and considering that women would be admitted to the hospital on the same day to undergo other procedures, we performed all the scheduled consultations as face‐to‐face visits. As for post‐test counseling, we decided upon a face‐to‐face consultation in case of pathological results, opting for a telemedicine meeting to return normal results, subject to patient's agreement. In our department, postnatal genetic activity is divided into outpatient clinic, for both first and follow‐up visits, and inpatient consultations, generally prompted by other specialists’ requests. Given the rapid spread of SARS‐CoV‐2 virus infection in Tuscany, all outpatient first visits were suspended from March 9th. The subsequent need to reschedule so many appointments has meant a significant organizational issue, which will probably require us to establish criteria in order to stratify consultations, based on their urgency and on the possibility to perform them as telemedicine visits. Concerning already scheduled outpatient follow‐up appointments, in these last two months we had to go through our working plan case‐by‐case, so to decide which ones could be performed as telemedicine consultations and which ones actually required an in‐person visit, rescheduling these last ones. Interestingly, once called by our medical staff, most of our patients’ parents (75%) told us that they would have cancelled the appointment themselves, fearing to contract COVID‐19 inside the hospital. We also opted for telemedicine consultations to return genetic tests results: following every remote consultation, we drafted a detailed report and sent it by e‐mail protected by a password communicated exclusively to the patient's family/caregiver.

Out of 90 telemedicine consultations performed (82 postnatal follow‐up visits and 8 prenatal consultations to return negative results), 84 (93%) families responded to the survey, reporting a satisfying level of communication.

With regard to inpatients’ genetic consultations, we observed a reduction in the number of requests received by our department in this period; we usually perform a minimum of 50 inpatient visits a month, but only 25 consultations have been requested from March 9th to April 30th. This noticeable decrease in the number of inpatient consultations reflects the reduction of routinely elective clinical and surgical activity in our hospital in this period. As reported in Figure 1, we performed all the inpatients consultations as face‐to‐face ones.

## 4 DISCUSSION

Fast spread of SARS‐CoV‐2 virus infection in Italy prompted our hospital to rapidly reorganize clinical and surgical activities, in order to optimize healthcare resources and minimize the risk of in‐hospital infections (Cini et al., 2020). Therefore, each department in our hospital was asked to temporarily suspend non‐urgent clinical and surgical procedures, to avoid unnecessary hospitalizations and outpatient admissions. To the best of our knowledge, this is the first report on changes in daily clinical practice at a medical genetic department of a tertiary pediatric hospital, during actual pandemic. Concerning the medical genetics department activity, we continued to perform prenatal first appointments and inpatient consultations as face‐to‐face visits, as patients would have been present in the hospital to perform other procedures in addition to our consult. Regarding prenatal care, technological advances in the field of prenatal genetic diagnosis have made it essential to provide detailed pre‐ and post‐test counseling to women and their families; current advanced molecular and cytogenetics technologies like SNP‐array and exome sequencing, which should be offered in a tertiary care centre, can reveal even unknown or of uncertain significance results which must be explained in detail by a medical geneticist (Hui, Szepe, Halliday, & Lewis, 2020).

Therefore, in addition to an extensive illustration of possible results and clinical implications of a given genetic test, it is important to provide support to patients and to reassure them (Harding, Hammond, Chitty, Hill, & Lewis, 2020). Even in such critical period, thanks to a well‐established prenatal diagnostic program that gives us the chance to concentrate multiple visits in a single day, our patients could benefit from face‐to‐face genetic consultations, receiving adequate support and gaining proper understanding of given information.

Regarding the postnatal setting, we maintained very few of the scheduled follow‐up appointments, choosing to either postpone or convert to telemedicine consultations most of them. We opted for telecommunication in selected cases, thus finding an efficient alternative to the classic face‐to‐face visit for ensuring continuity of care. Telemedicine consultation proved to be an effective method to provide genetic counseling, receiving positive feedback from both patients and genetics specialists: in a systematic review (Hilgart, Hayward, Coles, & Iredale, 2012), authors concluded that telegenetics, through the use of videoconferencing tools, is an acceptable method of performing any type of genetic consultation, both in the prenatal setting and to evaluate pediatric patients with suspected genetic conditions.

Previously, some experiences about the use of telegenetics across United States and Europe have been reported; however, application of telemedicine appeared to be different among the various hospitals (Otten, Birnie, Lucassen, Ranchor, & Van Langen, 2016; Terry et al., 2019). So far, in Italian healthcare, patients have never been inclined to accept telemedicine as a clinical procedure equivalent to in‐person visit: this uncertainty regarding the clinical value of telemedicine was probably related to the stereotype for which the development of a relationship between patient and physician could not occur without a face‐to‐face method.

Despite this reluctance to employ a method already in use in other European countries and so sporadically in our territory, the provision of support through a telehealth approach has been really useful to maintain the psychological well‐being of our patients.

According to our recent experience, telemedicine made it possible to remain in contact with patients, to follow and to answer questions about the clinical course of a given condition and to communicate genetic tests’ results providing an exhaustive explanation. So far, we opted for telemedicine only in selected post‐test consultations in the prenatal setting, and in case of follow‐up consultations in the postnatal setting, suspending all the first outpatient appointments for these two last months. Given the uncertainty of the current pandemic situation, it is now mandatory for us to think about how to deal with to the increasing demand for genetic counseling, also taking benefit from telemedicine.

**Figure 2:**
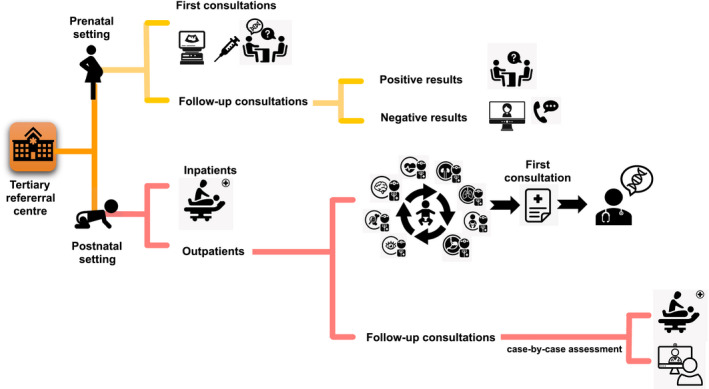
Reorganization of medical genetics department in the light of COVID‐19 pandemic

Since there is currently no standardized approach or guidelines on telegenetics, we need to establish specific criteria to make an *a priori* assessment of the first visits in terms of urgency, also trying to discern whether they can be performed as telemedicine consultation or must be done as in‐person visit. Being a tertiary referral centre, our hospital mostly attracts pediatric patients who need a multidisciplinary diagnostic approach, within which geneticist has the role to coordinate various investigations: in this specific setting, the experience of the clinical geneticist is essential and, usually, a detailed face‐to‐face evaluation is pivotal to guide the diagnostic path in order to reach the diagnosis (Malinowski et al., 2020). Since genetic consultations in pediatric patients are mainly required by pediatricians or other specialists, setting up a network for sharing patients’ clinical information might be extremely useful in order to stratify requests for first consultations. By adopting this approach, it would be possible to refer complex patients to a tertiary hospital, providing them with a well‐structured diagnostic path that includes a first face‐to‐face genetic consultation (Figure 2); instead, first‐line tests might be offered to patients with less complex and heterogeneous phenotypes in smaller hospitals.

In conclusion, spread of SARS‐CoV‐2 virus infection in Italy forced us to change our working habits. With regard to prenatal care, we opted for in‐person consultations, for they are scheduled on the same day of other procedures and also because we prefer to maintain a face‐to‐face approach in this delicate setting. As for postnatal care, we experienced satisfactory communication through telemedicine, for both returning genetic test results and receiving updates about our patients. Although we have always been used to a face‐to‐face counseling, the current pandemic situation made us get acquainted with the benefits of telemedicine in such a complex situation, also in terms of reducing waiting times.

Furthermore, given the limited number of clinical geneticists in our country, telegenetics services, which make genetic counseling feasible through the internet, can provide consultations faster and with a reduction of waiting lists. We experienced that genetic consultations performed with telemedicine revealed to be equal to face‐to face visits. Both patients and providers reported a greater appreciation of real‐time videoconferencing over telephone call to perform the consultation. It is interesting to note that, in addition to the fear of contracting an infection in the hospital, most families appreciated the proposal for telemedicine consultation because they could carry out the visit without travelling long distances.

Given the uncertainty of COVID‐19 outbreak's course, we must define a new plan to manage postnatal first consultations: developing a network to share clinical information among geneticists, pediatricians and other pediatric specialists might represent an essential step to prioritize the increasing number of requests, in order to choose wisely which cases should be approached with an in‐person genetic consultation in a multidisciplinary setting. Improvement of real‐time videoconferencing with high‐speed technologies will hopefully help us integrate telegenetics consultations in our clinical routine and will allow us to tele‐communicate even more complex and clinically significant results, providing a satisfying connection between patient and physicians.

## CONFLICT OF INTEREST

Nothing to disclose.

## AUTHOR CONTRIBUTION

PA, GS designed and conceived the project. PA, FG, DGF, GG, TG collected and analyzed data and survey results; PA, GS wrote the manuscript; IA, MG revised the manuscript; and GS supervised the project.
